# Supplementary material for: Automated segmentation of the larynx on computed tomography images: a review
Source: Biomed Eng Lett. 2022 Mar 18;12(2):175–83. doi: 10.1007/s13534-022-00221-3 (PMC9046475; doi:10.1007/s13534-022-00221-3)
Supplement: Supplementary file 1 — Supplementary Material 1 [file 13534_2022_221_MOESM1_ESM.docx]

Automated Segmentation of the Larynx on Computed Tomography Images: A Review

Divya Rao^1,2^, Prakashini K^3^, Rohit Singh^2*^ and Vijayananda J^4^

^1^ Department of Information and Communication Technology, Manipal Institute of Technology, MAHE, Manipal -576104, India.

^2^ Department of Otorhinolaryngology, Kasturba Medical College, MAHE, Manipal -576104, India.

^3^ Department of Radiodiagnosis and Imaging, Kasturba Medical College, MAHE, Manipal -576104, India.

^4^ Data Science and Artificial Intelligence, Philips,Bangalore-560045, India.

*Corresponding author. E-mail(s): [rohit.singh@manipal.edu](mailto:rohit.singh@manipal.edu);

Contributing authors: [divya.r@manipal.edu](mailto:divya.r@manipal.edu) ; [prakashini.k@manipal.edu](mailto:prakashini.k@manipal.edu) ; [vijayananda.j@philips.com](mailto:vijayananda.j@philips.com) ;

**Abstract:**

Larynx is an organ in the Head and Neck region. Larynx is one of the most common site of occurrence of Head and neck cancers. Segmentation of laryngeal tumors and substructures in imaging plays a key role in the diagnosis and staging of laryngeal cancer. Manual segmentation of the region of interest is time consuming. Reliable computer aided intervention in the contouring and segmentation process will help clinicians to easily verify their findings and look for oversight in diagnosis. The larynx is comparatively less explored in the head and neck region owing to the complex anatomical structure. Though there is a lot of emphasis on brain and lung in terms of automation in tumor detection and segmentation, the larynx organ has not received the same focus. Therefore, this work looks into all the research that has been done in automated segmentation, specifically of larynx anatomy in contrast computed tomography images. The publicly available datasets have been listed, different segmentation approaches, each with their own merits have been compared in our paper. We also summarize the performance of commercial medical contouring software that have ventured into this space. This work is useful for research that works with artificial intelligence in head and neck cancer, specifically that works with the segmentation of laryngeal anatomy.

Keywords: Larynx Segmentation, Artificial Intelligence, Medical Image Processing, Computed Tomography

# Introduction

Larynx is an organ that extends from the base of the tongue till the cricoid cartilage, before the start of the trachea. Commonly known as the voice box, it is a tube-shaped organ roughly 5 cm in length. It plays an important role in respiration, phonation, airway protection and swallowing. The larynx consists of bone, muscles, cartilage and a mucosal lining.

Larynx is one of the most common sites of occurrence of head and neck cancers. Consumption of alcohol and smoking are the major etiologic factors that contribute to laryngeal cancer [1]. More than 180,000 people worldwide are diagnosed with Laryngeal cancer every year [2]. As it has a poor prognosis, early detection and diagnosis and effective treatment is essential for better outcomes.

The larynx has three main subsites: the supraglottis, the glottis and the subglottis. Cancer can develop in any or all the subsites of the larynx. The incidence across subsites is not uniform: Incidence of cancer at the supraglottis is 30-50% of all cases, glottis is 50-70% while subglottis incidence is very rare at 0-1% of all laryngeal cancers. [4]

The Tumor-Node-Metastasis (TNM) staging system is a standard followed by the American Joint Committee on Cancer that describes the location of the cancer, the extent of its spread and if other parts of the body have been affected.


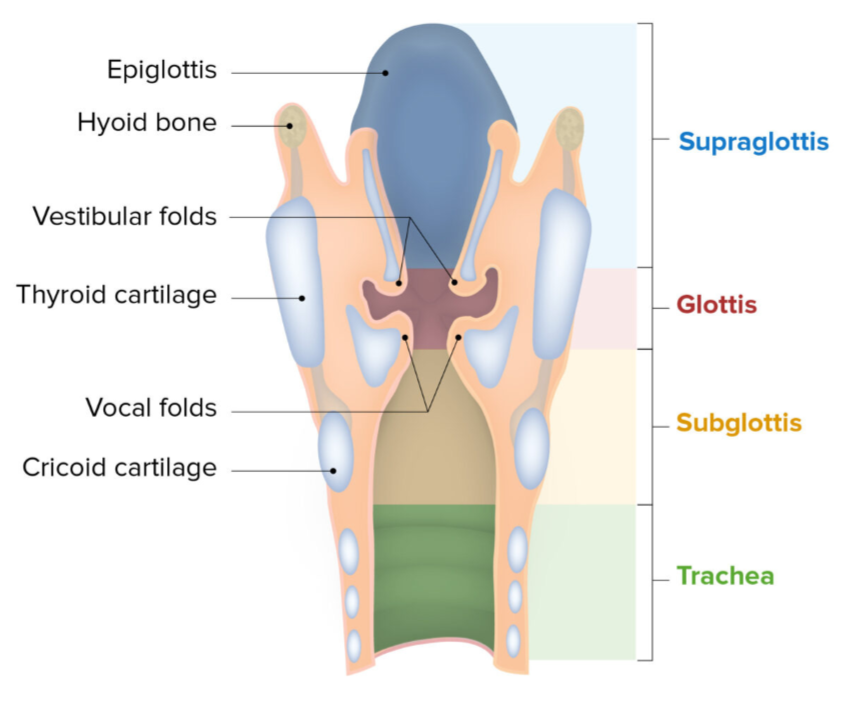


**Fig. 1**: Laryngeal anatomy and its subsites [3]


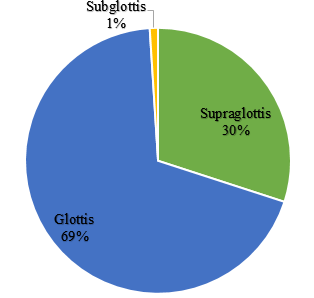


**Fig. 2**: Incidence of Laryngeal cancer across its sub sites

The T-staging is subsite specific and the number assigned is dependent on the spread of the tumor within the specific subsite impacted. The N-staging is common for the Larynx and reports the impact on the Lymph nodes. The M-number expresses if the cancer has metastasized [5]. Based on all the information collected, a treatment plan is designed for the patient. The treatment may be a combination of surgery, radiation therapy and chemotherapy.

Cancers diagnosed in the early stages have a high chance of preservation of the larynx and are highly curable. In contrast late stage cancers often require total laryngectomy and impact the quality of life of the patient. [6]

Imaging is useful in determining the extent of disease. In the early stages where the nodule is very small, neck imaging may be waived if surgery is not a mode of treatment. However, for advanced stages, a computed tomography or Magnetic Resonance Imaging (MRI) scan is administered to determine the invasion and spread of tumor and invasion of cancerous tissue. Contrast Computed Tomography (CT) imaging is useful in assessing the extent of cartilage


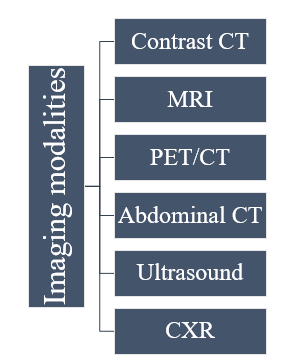


**Fig. 3**: Imaging modalities used to investigate the spread of laryngeal cancer

Invasion and submucosal disease. MRI with contrast is useful to determine pre epiglottic or paraglottic space invasion. It is important to note that over staging and under staging of disease are both detrimental to the treatment as the former will cause removal of healthy tissue, decreasing larynx function while the latter will leave behind cancerous tissue at the site.

Most common sites of distal metastasis for laryngeal cancer are the lungs followed by the liver. Chest X-ray (CXR) is used to investigate for distant metastasis of laryngeal cancer. Abdominal CT or liver Ultrasound scans are done if there is increased suspicion.

Positron emission tomography (PET)/CT is another imaging modality but its relevance in use of diagnosis is still debated. A PET is helpful in detecting subtle metabolically active lesions. Currently, a standard on imaging for laryngeal cancer imaging does not exist and the individual cases determine the preference of imaging modality selection. [7]

This paper focuses on the work that has been done in automating segmentation of the larynx and tumors in the larynx on CT images. The reason for choosing this modality for this review is that CT remains the more widely used imaging modality. CT is often used more widely as it is readily available, relatively cheaper than MRI and is rapidly acquired. MR has a greater cost for acquisition in terms of time, computation and resources. Optimization of MR techniques is difficult in this complex part of the body[7], It is much faster to capture a CT image( 60 seconds) while giving comparable results to MRI which takes longer to capture( 30 minutes). CT therefore requires less computation resources while compared to an MRI. The short capture time causes clearer images to be obtained and is not affected by patient movements such as coughing, sneezing, breathing etc. [8]

# Importance of Segmentation

Segmentation is localizing and delineating anatomical structures and tumors in a medical image. Early cancer detection works toward preventing advancement to further stages, increasing chances of a complete recovery. Under staging a tumor can cause parts of the cancerous tissue to be left behind after treatment. However, if a tumor is overstaged, this could lead unnecessary loss of healthy tissue, thereby limiting laryngeal function instead of preservation [9]. It is of utmost importance to accurately identify the cancerous tissue and plan the course of treatment action.

In this paper, we focus on the CT imaging modality. Contrast enhanced CT images are acquired for the larynx to look for tumor. Segmentation of CT images provide spatial and contextual information that is very valuable as it simplifies analysis and other follow up tasks such as treatment planning and TNM staging.

The T stage is dependent on the affected anatomies that may show up only in a single slice, depending on the thickness of the CT image. The size of the tumor and the anatomies affected are crucial pieces of information that determine the stage, which in turn determines the treatment course.

There are a few challenges in using the CT imaging modality for laryngeal cancer diagnosis. In conventional contrast CT images, the movement of vocal cords is not captured. This is an important parameter in the T staging of Glottic tumors. This has to be observed via clinical reports or a phonation CT image has to be captured. Iodine-enhanced tumors and non-ossified cartilage are challenging to distinguish. Also, cartilage invasion can cause over-staging as it can appear indistinguishable to the human eye [10].

# Datasets In this area

**Table I**: Datasets available

| **Ref.** | **Size** | **CT image** | **Segmentation** | **Availability** |
| --- | --- | --- | --- | --- |
| 1 [11] | 45 | Contrast Enhanced Volume | Larynx | Not Public |
| 2 [11] | 32 | Contrast Enhanced Volume | Larynx | Not Public |
| 3 [12] | 265 | Contrast Enhanced Volume | Tumor | On Request |
| 4 [13] | 326 | Harmonized slice | Presence of  Abnormality | Not Public |
| 5 [14] | 606 | Contrast Enhanced Volume | Tumor | Not Public |
| 6 [15] | 241 | Contrast Enhanced Volume | Tumor | Not Public |
| 7 [16] | 185 | Contrast Enhanced Volume | Larynx | Not Public |
| 8 [17] | 1160 | Non Contrast Enhanced Volume | Larynx | Not Public |
| 9 [18] | 364 | Contrast Enhanced Volume | Larynx | On Request |
| 10 [19] | 36 | Contrast Enhanced Volume | Tumor | Public |
| 11 [20] | 6 | Contrast Enhanced Volume | Tumor | Public |
| 12 [21] | 42 | Contrast Enhanced Volume | Tumor | Public |

# Automated Segmentation

An automated segmentation of sub-anatomical structures and lesions on the imaging data is useful in saving the detection and diagnostic time of the radiologist and the clinician.

Manual outlining of the larynx Region of interest (ROI) by a radiologist can take up to 2.5 hours for a single CT image series [22].

There are various approaches to automate the segmentation process.

## Atlas Based Segmentation

Atlas based segmentation involves using a reference image to indicate prior knowledge to help with the segmentation task. Atlas based methods are widely used in medical image processing. [23]


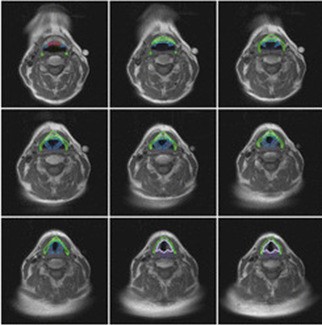


**Fig. 4**: Atlas based registration of laryngeal anatomic substructures [24]

## Supervised and Unsupervised Image Segmentation

Supervised segmentation algorithms use a set of labelled images, which are pre categorized data, with apriori knowledge and human input for designing the segmentation model. They are quite powerful for automatic segmentation of medical images albeit not versatile when there is a lot of variance between the train and test sets. They use classification and regression techniques for the segmentation challenge.

Unsupervised Image segmentation algorithms work with unlabeled data and work by generating a split between non-homogeneous regions into various sub-regions using statistical parameters of the image. Clustering, Association and Dimensionality reduction are commonly used techniques in this approach.

## Convolutional Neural Networks

Convolutional Neural Network (CNN) is an approach uses deep learning. The architecture of a CNN consists of layers of interconnected nodes, with assigned weights that get updated when the model is trained. Pairs of images with the expected segmentations are taken as input to train the model which learns to segment the ROI by assigning weights to various aspects or objects in the given input images. The final trained model uses these weights to compute the segmentation of the input images. 3D CNNs can capture spatial dependencies on CT images because of the architecture and through the application of

Relevant filters


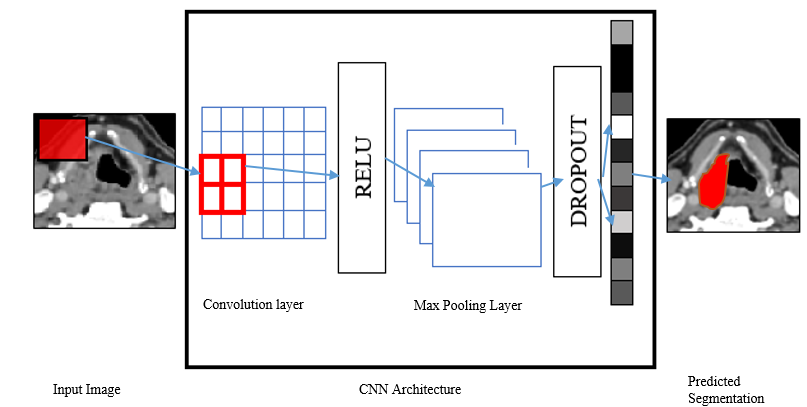


**Fig. 5**: CNN architecture

# Automatic Segmentation of the Larynx

Various techniques using atlas based registration technique were attempted for outlining the laryngeal contours.

Mencarelli et al [25] designed a hierarchical model to represent substructures and then used unsupervised learning to detect substructures. The automatic segmentation was successful in 88% of the time for identifying the ROI of the larynx without manual intervention. However, they used Bland Altman method instead of the more straightforward Dice Similarity Index (DSC) approach for checking overlap between the expected segmentations and the results obtained. Wu et al [26] used fuzzy models and worked on the hierarchy based on relationships between detected objects and a delineation algorithm to contour the substructures of head and neck CT images. Thomson et al [27], reported results obtained with an atlas based approach that was statistically significantly worse than Mencarelli’s [25] model. Only 8% of the larynx contours could be used as is, without alterations. With introduction of bias in their algorithm, they were able to get the DSC significantly higher, to 84%.

Another atlas based approach [11] that used a consensus voting scheme to contour obtained a DSC of 71% for the larynx. Tao et al [28] used a combination of manual delineation and atlas based auto segmentation to get a DSC of 73% for the supraglottis and 64% for the glottis segmentation.

| Year | Author | Anatomy | DSC | Method | Dataset |
| --- | --- | --- | --- | --- | --- |
| 2014 | Mencarelli[25] | Larynx | - | Multi AB Registration | 188 |
| 2014 | Thompson et al. [27] | Larynx | incomparable | AB and Model based | 10 |
| 2014 | Thompson et al. [27] | Larynx | 84% | AB and Model based with intervention | 10 |
| 2015 | Tao et al. [28] | Supraglottis | 73% | Multi AB Registration | 16 |
| 2015 | Tao et al. [28] | Glottis | 64% | Multi AB Registration | 16 |
| 2017 | Ibragimov et al [29] | Larynx | 85% | CNN | 45 |
| 2018 | Wu et al [26] | Larynx | 74% | Fuzzy Model | 216 |
| 2018 | Willems et al. [35] | Larynx | 39% | CNN | 90 |
| 2019 | Haq et al. [11] | Larynx | 71% | Multi AB Registration | 77 |
| 2019 | van Rooij et al. [30] | Larynx | 78% | DLC | 136 |
| 2019 | Liang et al. [16] | Larynx | 87% | CNN | 185 |
| 2020 | van Dijk et al. [31] | Larynx | 71% | DLC | 311 |
| 2020 | Lei et al. [34] | Larynx | 83% | URCNN | 15 |
| 2021 | Fang et al. [17] | Larynx (non enhanced CT) | 74% | UNet | 800 |
| 2021 | Zhong et al. cite[18] | Larynx | 84% | CNN | 364 |
| 2021 | Soomro et al [33] | Larynx | 80% | DenseNet | 46 |

AB: Atlas Based, CNN: Convolutional Neural Networks, DLC: Deep Learning Contouring

Table II: Summary of Approaches to the Automatic Segmentation of the Larynx using CT images

Ibragimov and Xing [29] were the one of the first to use deep learning methods for larynx segmentation in Head-and-Neck CT images. They used a CNN to auto-segment the larynx using a 45 image dataset to arrive at an 85.6± 4.2% DSC. Rooij et al [30] used the 3D-Unet architecture to auto-segment the larynx in a deep learning approach. They produced DSC of 0.71 ± 0.10 for the larynx with the largest dataset of 311 images all the studies surveyed.

Liang et al [16] developed the ODS net and obtained a dice score for larynx of 87% with a dataset of 185 images. They used a modified version of fully convoluted neural network. Zhong et al [18] used a variation of UNet on a dataset of 364 images and obtained a dice score of 84%. Tam et al [32] employed an architecture that exploited shape features and a multiple output support vector model with regression techniques. OARNet [33] with 235 images were able to reach a DSC of 80%. They employed a dense CNN for drawing a bounding box and then segmented the ROI. Lei et al [34] with a goal of reduction in computation time, constructed a recurrent CNN with a U structure and were able to segment the larynx ROI with a DSC of 83% with 15 larynx images.


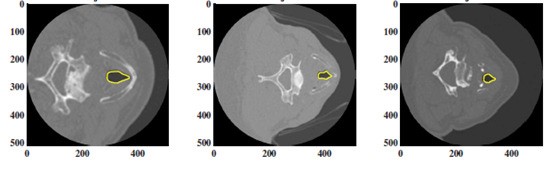


**Fig. 6**: Rimaglottidis segmentation output [36]

## Commercial contouring software

There are a few commercial contouring softwares available. A thorough comparison has been carried out [22], regarding the efficiency of these algorithms in auto contouring. The larynx by these softwares. After altering the contours manually, they gave a higher DSC score.

**Table 3**: Commercial Auto Segmentation software results on Larynx CT images

| Commercial Software | Automatic DSC | DSC after manual correction |
| --- | --- | --- |
| ABAS 2.0 | 86% | 90% |
| MIM 5.1.1 | 87% | 89% |
| Velocity AI 2.6.2 | 82% | 86% |

## Relevant Notable Work

Hewavitharanage et al. [36] developed an auto segmentation algorithm to segment a single anatomical substructure of the larynx using GLDM texture features and support vector machines. With a sample size of 20 subjects, they managed to get an over 80% area overlap with the manual annotations. In another paper, the author reported developing an algorithm to determining the slice of CT which contains the vocal fold by localization of the vertebral column and anterior commissure [37]. Using a combination of techniques, they achieved an 85% accuracy in estimation in their 20 patients.

Santin et al [13] used deep learning to detect the presence of cartilage abnormalities in CT Scans. With a dataset of 326 images, they obtained an AUC of .72 in their binary classification problem that used an adapted VGG16 network with good sensitivity (83

The best accuracy reached in a thyroid cartilage classification challenge [14] as normal or abnormal, with a total of 511 images was an AUC of 70%. However, details of the model used were not disclosed in the paper. Ayyalu et al [38] worked on checking the dependence of anatomic similarity for Auto Segmentation of Head and Neck CT images. With ten patients captures in different scenarios ranging from poor to perfect, they used multiple atlases to perform auto contouring. They concluded that larynx auto segmentation was heavily dependent on anatomic similarity compared to the other organs.

Daix et al [39] worked on auto segmentation organs in the head and neck using dual pyramid networks and obtained a DSC of 90% for cone beam CT images. There are other works [40],[41],[42],[43],[15],[44] that are have appreciable models for automated segmentation of head and neck organs at risk that did not include the larynx.

# Discussion and Conclusion

There has been notable work done in the segmentation of the larynx and substructures. The studies however have been limited, with many studies using a small number of images. The availability of segmented larynx CT datasets in the public domain is almost negligible. It is therefore difficult to develop and validate automated segmentation models for independent researchers to work with.

There are a few key challenges for automated segmentation of the larynx on contrast CT images. Firstly, there is a lack of a collective standard for segmentation of the larynx. Ground truth creation is necessary and uniformity is desired for a large dataset for the auto segmentation model to perform with acceptable sensitivity and specificity. It can be challenging for the radiographer to differentiate between lesion and swollen tissue in certain scenarios. Contrast settings of CT scanners used during image acquisition and thickness of CT slices may vary across centers. This calls for standardization of CT images during acquisition. As we noted earlier, the modality is decided based on the case of the patient as MR may be preferred over CT for some cases.

It is also noted that there is a lot of work done in deep learning, auto contouring, and segmentation in the Head and Neck CT images with promising results. However, the brain stem, spinal cord remains the most studied organs at risk. Most head and neck delineation studies tend to leave out the larynx. We argue that there is a great need for more research in this area as it standardization and auto segmentation can lead to more effective treatment. Auto segmentation, if implemented usefully of the larynx can be a helpful tool for better, easier and quicker diagnosis radiotherapy planning.

# Statements and Declarations

This work has been funded by Philips Innovation Campus, Bangalore and carried out jointly by Philips Innovation Campus, Bangalore and Kasturba Medical College, Manipal. (Exhibit B-027 of INR450000)

The authors have no conflict of interest to declare.

# References

1. Muscat, J.E., Wynder, E.L.: Tobacco, alcohol, asbestos, and occupational risk factors for laryngeal cancer. Cancer **69** (1992). [https://doi.org/10.1002/1097-0142(19920501)69:9h2244:: AID-CNCR2820690906i3.0.CO;2-O](https://doi.org/10.1002/1097-0142(19920501)69:9%3c2244::AID-CNCR2820690906%3e3.0.CO;2-O)
2. Anon: Larynx Gco.iarc.fr

(2020). [https://gco.iarc.fr/today/data/factsheets/cancers/](https://gco.iarc.fr/today/data/factsheets/cancers/14-Larynx-fact-sheet.pdf)

[14-Larynx-fact-sheet.pdf](https://gco.iarc.fr/today/data/factsheets/cancers/14-Larynx-fact-sheet.pdf) Accessed 2021-10-08

1. Anon: Lecturio. 2021. Larynx Concise Medical Knowledge.

<https://www.lecturio.com/concepts/larynx/>

1. Siegel, R.L., Miller, K.D., Fuchs, H.E., Jemal, A.: Cancer statistics, 2021. CA: A Cancer Journal for Clinicians **71** (2021). [https://doi.org/10.3322/ caac.21654](https://doi.org/10.3322/caac.21654)
2. Amin, M., Byrd, D., Edge, S., Greene, F.: AJCC Cancer Staging Manual. Springer, ??? (2016)
3. Issa, M., Samuels, S., Bellile, E., Shalabi, F., Eisbruch, A., Wolf, G.: Tumor volumes and prognosis in laryngeal cancer. Cancers **7** (2015). <https://doi.org/10.3390/cancers7040888>
4. Junn, J.C., Soderlund, K.A., Glastonbury, C.M.: Imaging of head and neck cancer with ct, mri, and us. Seminars in Nuclear Medicine **51** (2021). <https://doi.org/10.1053/j.semnuclmed.2020.07.005>
5. Adolphs, A.P.J., Boersma, N.A., Diemel, B.D.M., Eding, J.E.C., Flokstra, F.E., Wegner, I., Grolman, W., Braunius, W.: A systematic review of computed tomography detection of cartilage invasion in laryngeal carcinoma. The Laryngoscope **125** (2015). <https://doi.org/10.1002/lary.25145>
6. Li, B., Bobinski, M., Gandour-Edwards, R., Farwell, D.G., Chen, A.M.: Overstaging of cartilage invasion by multidetector ct scan for laryngeal cancer and its potential effect on the use of organ preservation with chemoradiation. The British Journal of Radiology **84** (2011). [https: //doi.org/10.1259/bjr/66700901](https://doi.org/10.1259/bjr/66700901)
7. Kuno, H., Sakamaki, K., Fujii, S., Sekiya, K., Otani, K., Hayashi, R., Yamanaka, T., Sakai, O., Kusumoto, M.: Comparison of mr imaging and dual-energy ct for the evaluation of cartilage invasion by laryngeal and hypopharyngeal squamous cell carcinoma. American Journal of Neuroradiology **39** (2018). <https://doi.org/10.3174/ajnr.A5530>
8. Haq, R., Berry, S.L., Deasy, J.O., Hunt, M., Veeraraghavan, H.: Dynamic multiatlas selection-based consensus segmentation of head and neck structures from ct images. Medical Physics **46** (2019). [https://doi.org/10.1002/ mp.13854](https://doi.org/10.1002/mp.13854)
9. Guo, R., Guo, J., Zhang, L., Qu, X., Dai, S., Peng, R., Chong, V.F.H., Xian, J.: Ct-based radiomics features in the prediction of thyroid cartilage invasion from laryngeal and hypopharyngeal squamous cell carcinoma. Cancer Imaging **20** (2020). <https://doi.org/10.1186/s40644-020-00359-2>
10. Santin, M., Brama, C., Th´ero, H., Ketheeswaran, E., El-Karoui, I., Bidault, F., Gillet, R., Teixeira, P.G., Blum, A.: Detecting abnormal thyroid cartilages on ct using deep learning. Diagnostic and Interventional

Imaging **100** (2019). <https://doi.org/10.1016/j.diii.2019.01.008>

1. Lassau, N., Estienne, T., de Vomecourt, P., Azoulay, M., Cagnol, J., Garcia, G., Majer, M., Jehanno, E., Renard-Penna, R., Balleyguier, C., Bidault, F., Caramella, C., Jacques, T., Dubrulle, F., Behr, J., Poussange, N., Bocquet, J., Montagne, S., Cornelis, F., Faruch, M., Bresson, B., Brunelle, S., Jalaguier-Coudray, A., Amoretti, N., Blum, A., Paisant, A., Herreros, V., Rouviere, O., Si-Mohamed, S., Marco, L.D., Hauger, O., Garetier, M., Pigneur, F., Berg`ere, A., Cyteval, C., Fournier, L., Malhaire, C., Drape, J.-L., Poncelet, E., Bordonne, C., Cauliez, H., Budzik, J.-F., Boisserie, M., Willaume, T., Moli`ere, S., Faure, N.P., Giurca, S.C., Juhan, V., Caramella, T., Perrey, A., Desmots, F., Faivre-Pierre, M., Abitbol, M., Lotte, R., Istrati, D., Guenoun, D., Luciani, A., Zins, M., Meder, J.-F., Cotten, A.: Five simultaneous artificial intelligence data challenges on ultrasound, ct, and mri. Diagnostic and Interventional Imaging **100** (2019). <https://doi.org/10.1016/j.diii.2019.02.001>
2. Liu, X., Maleki, F., Muthukrishnan, N., Ovens, K., Huang, S.H., P´erezLara, A., Romero-Sanchez, G., Bhatnagar, S.R., Chatterjee, A., Pusztaszeri, M.P., Spatz, A., Batist, G., Payabvash, S., Haider, S.P., Mahajan, A., Reinhold, C., Forghani, B., O’Sullivan, B., Yu, E., Forghani, R.: SiteSpecific Variation in Radiomic Features of Head and Neck Squamous Cell Carcinoma and Its Impact on Machine Learning Models (2021). <https://doi.org/10.3390/cancers13153723>
3. Liang, S., Tang, F., Huang, X., Yang, K., Zhong, T., Hu, R., Liu, S., Yuan, X., Zhang, Y.: Deep-learning-based detection and segmentation of organs at risk in nasopharyngeal carcinoma computed tomographic images for radiotherapy planning. European Radiology **29** (2019). [https: //doi.org/10.1007/s00330-018-5748-9](https://doi.org/10.1007/s00330-018-5748-9)
4. Fang, Y., Wang, J., Ou, X., Ying, H., Hu, C., Zhang, Z., Hu, W.: The impact of training sample size on deep learning-based organ autosegmentation for head-and-neck patients. Physics in Medicine Biology **66** (2021). <https://doi.org/10.1088/1361-6560/ac2206>
5. Zhong, Y., Yang, Y., Fang, Y., Wang, J., Hu, W.: A preliminary experience of implementing deep-learning based auto-segmentation in head and neck cancer: A study on real-world clinical cases. Frontiers in Oncology **11** (2021). <https://doi.org/10.3389/fonc.2021.638197>
6. Bosch, W.R., Straube, W.L., Matthews, J.W., Purdy, J.A.: Head-neck cetuximab - the cancer imaging archive (2015). [https://doi.org/10.7937/ K9/TCIA.2015.7AKGJUPZ](https://doi.org/10.7937/K9/TCIA.2015.7AKGJUPZ)
7. Zuley, M.L., Jarosz, R., Kirk, S., Y., L., Colen, R., Garcia, K., Aredes, N.D.: Radiology data from the cancer genome atlas head-neck squamous cell carcinoma [TCGA-HNSC] collection (2016). [https://doi.org/10.7937/ K9/TCIA.2016.LXKQ47MS](https://doi.org/10.7937/K9/TCIA.2016.LXKQ47MS)
8. Brouwer, C.L., Steenbakkers, R.J.H.M., Bourhis, J., Budach, W., Grau, C., Gr´egoire, V., van Herk, M., Lee, A., Maingon, P., Nutting, C., O’Sullivan, B., Porceddu, S.V., Rosenthal, D.I., Sijtsema, N.M., Langendijk, J.A.: Ct-based delineation of organs at risk in the head and neck region: Dahanca, eortc, gortec, hknpcsg, ncic ctg, ncri, nrg oncology and trog consensus guidelines. Radiotherapy and Oncology **117** (2015). <https://doi.org/10.1016/j.radonc.2015.07.041>
9. Macchia, M.L., Fellin, F., Amichetti, M., Cianchetti, M., Gianolini, S., Paola, V., Lomax, A.J., Widesott, L.: Systematic evaluation of three different commercial software solutions for automatic segmentation for adaptive therapy in head-and-neck, prostate and pleural cancer. Radiation Oncology **7** (2012). <https://doi.org/10.1186/1748-717X-7-160>
10. Cuadra, M.B., Duay, V., Thiran, J.-P.: Atlas-based Segmentation. [https: //doi.org/10.1007/978-0-387-09749-7](https://doi.org/10.1007/978-0-387-09749-7_12) [12](https://doi.org/10.1007/978-0-387-09749-7_12)
11. Choi, M., Refaat, T., Lester, M.S., Bacchus, I., Rademaker, A.W., Mittal, B.B.: Development of a standardized method for contouring the larynx and its substructures. Radiation Oncology **9** (2014). [https://doi.org/10. 1186/s13014-014-0285-4](https://doi.org/10.1186/s13014-014-0285-4)
12. Mencarelli, A., van Kranen, S.R., Hamming-Vrieze, O., van Beek, S., Rasch, C.R.N., van Herk, M., Sonke, J.-J.: Deformable image registration for adaptive radiation therapy of head and neck cancer: Accuracy and precision in the presence of tumor changes. International Journal of Radiation Oncology*Biology*Physics **90** (2014). [https://doi.org/10. 1016/j.ijrobp.2014.06.045](https://doi.org/10.1016/j.ijrobp.2014.06.045)
13. Wu, X., Udupa, J.K., Tong, Y., Odhner, D., Pednekar, G., Simone, C.B., McLaughlin, D.J., Apinorasethkul, C., Shammo, G., Camaratta, J., Torigian, D.A., Lukens, J., Mihailidis, D., James, P.: Auto-contouring via automatic anatomy recognition of organs at risk in head and neck cancer on ct images. In: Webster, R.J., Fei, B. (eds.) SPIE, ??? (2018). <https://doi.org/10.1117/12.2293946>
14. Thomson, D., Boylan, C., Liptrot, T., Aitkenhead, A., Lee, L., Yap, B., Sykes, A., Rowbottom, C., Slevin, N.: Evaluation of an automatic segmentation algorithm for definition of head and neck organs at risk. Radiation Oncology **9** (2014). <https://doi.org/10.1186/1748-717X-9-173>
15. Tao, C.-J., Yi, J.-L., Chen, N.-Y., Ren, W., Cheng, J., Tung, S., Kong, L., Lin, S.-J., Pan, J.-J., Zhang, G.-S., Hu, J., Qi, Z.-Y., Ma, J., Lu, J.-D., Yan, D., Sun, Y.: Multi-subject atlas-based auto-segmentation reduces interobserver variation and improves dosimetric parameter consistency for organs at risk in nasopharyngeal carcinoma: A multi-institution clinical study. Radiotherapy and Oncology **115** (2015). [https://doi.org/10.1016/ j.radonc.2015.05.012](https://doi.org/10.1016/j.radonc.2015.05.012)
16. Ibragimov, B., Xing, L.: Segmentation of organs-at-risks in head and neck ct images using convolutional neural networks. Medical Physics **44** (2017). <https://doi.org/10.1002/mp.12045>
17. van Rooij, W., Dahele, M., Brandao, H.R., Delaney, A.R., Slotman, B.J., Verbakel, W.F.: Deep learning-based delineation of head and neck organs at risk: Geometric and dosimetric evaluation. International Journal of Radiation Oncology*Biology*Physics **104** (2019). [https://doi.org/ 10.1016/j.ijrobp.2019.02.040](https://doi.org/10.1016/j.ijrobp.2019.02.040)
18. van Dijk, L.V., den Bosch, L.V., Aljabar, P., Peressutti, D., Both, S., Steenbakkers, R.J.H.M., Langendijk, J.A., Gooding, M.J., Brouwer, C.L.: Improving automatic delineation for head and neck organs at risk by deep learning contouring. Radiotherapy and Oncology **142** (2020). [https: //doi.org/10.1016/j.radonc.2019.09.022](https://doi.org/10.1016/j.radonc.2019.09.022)
19. Tam, C., Tian, S., Beitler, J.J., Jiang, X., Li, S., Yang, X.: Automated delineation of organs-at-risk in head and neck ct images using multioutput support vector regression. In: Gimi, B., Krol, A. (eds.) SPIE, ??? (2018). <https://doi.org/10.1117/12.2292556>
20. Soomro, M.H., Nourzadeh, H., Alves, V.G.L., Choi, W., Siebers, J.V.: OARnet: Automated organs-at-risk delineation in Head and Neck CT images (2021)
21. Lei, Y., Harms, J.M., Dong, X., Wang, T., Tang, X., Yu, D.S., Beitler, J.J., Curran, W.J., Liu, T., Yang, X.: Organ-at-risk (oar) segmentation in head and neck ct using u-rcnn. In: Hahn, H.K., Mazurowski, M.A. (eds.) SPIE, ??? (2020). <https://doi.org/10.1117/12.2549782>
22. Willems, S., Crijns, W., Saint-Esteven, A.L.G., Veen, J.V.D., Robben, D., Depuydt, T., Nuyts, S., Haustermans, K., Maes, F.: Clinical Implementation of DeepVoxNet for Auto-Delineation of Organs at Risk in Head and Neck Cancer Patients in Radiotherapy (2018). [https://doi.org/10.1007/ 978-3-030-01201-4](https://doi.org/10.1007/978-3-030-01201-4_24) [24](https://doi.org/10.1007/978-3-030-01201-4_24)
23. Hewavitharanage, S., Gubbi, J., Thyagarajan, D., Lau, K., Palaniswami, M.: Automatic segmentation of the rima glottidis in 4d laryngeal ct scans in parkinson’s disease. IEEE, ??? (2015). [https://doi.org/10.1109/EMBC. 2015.7318468](https://doi.org/10.1109/EMBC.2015.7318468)
24. Hewavitharanage, S., Gubbi, J., Thyagarajan, D., Lau, K., Palaniswami,

M.: Estimation of vocal fold plane in 3d ct images for diagnosis of vocal

fold abnormalities. IEEE, ??? (2015). [https://doi.org/10.1109/EMBC. 2015.7319049](https://doi.org/10.1109/EMBC.2015.7319049)

1. Ayyalusamy, A., Vellaiyan, S., Subramanian, S., Ilamurugu, A., Satpathy, S., Nauman, M., Katta, G., Madineni, A.: Auto-segmentation of head and neck organs at risk in radiotherapy and its dependence on anatomic similarity. Radiation Oncology Journal **37** (2019). [https://doi.org/10.3857/ roj.2019.00038](https://doi.org/10.3857/roj.2019.00038)
2. Dai, X., Lei, Y., Wang, T., Dhabaan, A.H., McDonald, M., Beitler, J.J., Curran, W.J., Zhou, J., Liu, T., Yang, X.: Head-and-neck organs-at-risk auto-delineation using dual pyramid networks for cbct-guided adaptive radiotherapy. Physics in Medicine Biology **66** (2021). [https://doi.org/10. 1088/1361-6560/abd953](https://doi.org/10.1088/1361-6560/abd953)
3. Zhu, W., Huang, Y., Zeng, L., Chen, X., Liu, Y., Qian, Z., Du, N., Fan, W., Xie, X.: Anatomynet: Deep learning for fast and fully automated whole-volume segmentation of head and neck anatomy. Medical Physics **46** (2019). <https://doi.org/10.1002/mp.13300>
4. Vrtovec, T., Moˇcnik, D., Strojan, P., Pernuˇs, F., Ibragimov, B.: Autosegmentation of organs at risk for head and neck radiotherapy planning: From atlas-based to deep learning methods. Medical Physics **47** (2020). <https://doi.org/10.1002/mp.14320>
5. Fritscher, K.D., Peroni, M., Zaffino, P., Spadea, M.F., Schubert, R., Sharp, G.: Automatic segmentation of head and neck ct images for radiotherapy treatment planning using multiple atlases, statistical appearance models, and geodesic active contours. Medical Physics **41** (2014). [https://doi.org/ 10.1118/1.4871623](https://doi.org/10.1118/1.4871623)
6. Tong, N., Gou, S., Yang, S., Ruan, D., Sheng, K.: Fully automatic multiorgan segmentation for head and neck cancer radiotherapy using shape representation model constrained fully convolutional neural networks. Medical Physics **45** (2018). <https://doi.org/10.1002/mp.13147>
7. Nikolov, S., Blackwell, S., Zverovitch, A., Mendes, R., Livne, M., Fauw, J.D., Patel, Y., Meyer, C., Askham, H., Romera-Paredes, B., Kelly, C., Karthikesalingam, A., Chu, C., Carnell, D., Boon, C., D’Souza, D., Moinuddin, S.A., Garie, B., McQuinlan, Y., Ireland, S., Hampton, K., Fuller, K., Montgomery, H., Rees, G., Suleyman, M., Back, T., Hughes, C., Ledsam, J.R., Ronneberger, O.: Deep learning to achieve clinically applicable segmentation of head and neck anatomy for radiotherapy (2021)
