# Supplementary material for: Automated segmentation of the larynx on computed tomography images: a review
Source: Biomed Eng Lett. 2022 Mar 18;12(2):175–83. doi: 10.1007/s13534-022-00221-3 (PMC9046475; doi:10.1007/s13534-022-00221-3)
Supplement: Supplementary file 3 — Supplementary Material 3 [file 13534_2022_221_MOESM3_ESM.docx]

Automated Segmentation of the Larynx on Computed Tomography Images: A Review

Divya Rao^1,2^, Prakashini K^3^, Rohit Singh^2*^ and Vijayananda J^4^

^1^ Department of Information and Communication Technology, Manipal Institute of Technology, Manipal Academy of Higher Education, Manipal -576104, India.

^2^ Department of Otorhinolaryngology, Kasturba Medical College, Manipal Academy of Higher Education, Manipal -576104, India.

^3^ Department of Radiodiagnosis and Imaging, Kasturba Medical College, Manipal Academy of Higher Education, Manipal -576104, India.

^4^ Data Science and Artificial Intelligence, Philips, Bangalore-560045, India.

*Corresponding author. E-mail(s): [rohit.singh@manipal.edu](mailto:rohit.singh@manipal.edu); Contributing authors: [divya.r@manipal.edu](mailto:divya.r@manipal.edu) ; [prakashini.k@manipal.edu](mailto:prakashini.k@manipal.edu) ; [vijayananda.j@philips.com](mailto:vijayananda.j@philips.com) ; *^†^*These authors contributed equally to this work.

# Abstract

The larynx, or the voice-box, is a common site of occurrence of Head and Neck cancers. Yet, automated segmentation of the larynx has been receiving very little attention. Segmentation of organs is an essential step in cancer treatment-planning. Computed Tomography scans are routinely used to assess the extent of tumor spread in the Head and Neck as they are fast to acquire and tolerant to some movement.

This paper reviews various automated detection and segmentation methods used for the larynx on Computed Tomography images. Registration and deep learning approaches to segmenting the laryngeal anatomy are compared, highlighting their strengths and shortcomings. A list of available annotated laryngeal computed tomography datasets is compiled for encouraging further research. Commercial software currently available for larynx contouring are briefed in our work.

We conclude that the lack of standardisation on larynx boundaries and the complexity of the relatively small structure makes automated segmentation of the larynx on computed tomography images a challenge. Reliable computer aided intervention in the contouring and segmentation process will help clinicians easily verify their findings and look for oversight in diagnosis. This work is useful for research that works with artificial intelligence in Head and Neck cancer, specifically that works with the segmentation of laryngeal anatomy.

***Keywords***: Larynx Segmentation, Computer Aided Detection, Artificial Intelligence, Medical Image Processing, Computed Tomography

# Introduction

Commonly known as the voice box, the larynx is a tube-shaped organ roughly 5 cm in length. The larynx is responsible for speech, plays a vital role in respiration, and prevents food particles from entering the airway into the respiratory system. The larynx is one of the most common sites of occurrence of Head and Neck cancers [1]. Smoking tobacco and consumption of alcohol are the major etiologic factors that contribute to laryngeal cancer [2]. More than 180,000 people worldwide are diagnosed with Laryngeal cancer every year [3]. As it has a poor prognosis, early detection, diagnosis, and effective treatment are essential for better outcomes.

### The larynx extends from the base of the tongue to the cricoid cartilage before the start of the trachea. It is comprised of bone, muscles, cartilage, and a mucosal lining. The three main subsites of the larynx are the supraglottis, the glottis, and the subglottis as represented in Figure 1. Cancer can develop in any or all the subsites of the larynx. The incidence across subsites is not uniform: Cancer incidence at the supraglottis is 30-50% of all cases, the glottis is 50%-70%, and the subglottis incidence is scarce at 0-1% of all laryngeal cancers [4].


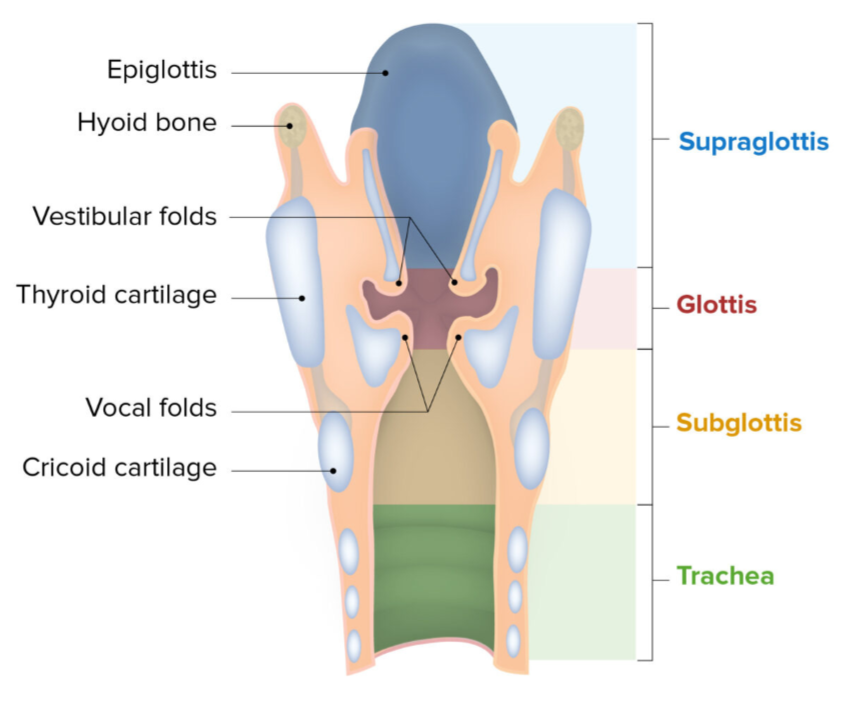


**Figure 1**: Laryngeal anatomy and its subsites [5].

When cancer is detected in the larynx, the Tumor-Node-Metastasis (TNM) staging system, a standard followed by the American Joint Committee on Cancer, is used to describe the malignant tumour. The TNM stage highlights the specific location of cancer within the larynx, the extent of its spread, and if other parts of the body have been affected. The T-staging is subsite specific and the T-number assigned depends on the spread of the tumour within the specific subsite impacted. The N-stage reports the impact of cancer on the lymph nodes of the entire larynx organ. The M-stage expresses if cancer has metastasized [6]. Based on the diagnostic and staging information collected, a treatment plan is designed for the patient. Depending on the severity of the case, the course of treatment may be a combination of surgery, radiation therapy, and chemotherapy. Cancers of the larynx diagnosed in the early stages have a high chance of organ preservation and are highly curable. In contrast, those diagnosed in advanced stages often require total laryngectomy, which has a severe impact on the quality of life of the patient [7] as it affects the day to day activities at both a physical and psychological level.

Imaging helps determine the extent of the disease. There are various radiographic imaging modalities that are commonly used to investigate the spread of disease as illustrated in Table I. It is important to note that over-staging and under-staging of disease are detrimental to treatment as the former will cause the removal of healthy tissue, decreasing larynx function, while the latter will leave behind cancerous tissue at the site. In the early stages of cancer, where the nodule is very small, neck imaging may be waived if surgery is not a mode of treatment. However, for advanced stages, Computed Tomography (CT) or Magnetic Resonance Imaging (MRI) scans are administered to determine the spread of tumour and invasion of cancerous tissue. Laryngeal cancer tends to metastasize in the lungs and the liver. Chest X-rays are used to investigate for such metastases. Additionally, but rarely Abdominal CT or liver Ultrasound scans are done to analyse the extent and spread of disease. Positron emission tomography (PET)/CT is another imaging modality, but its relevance in Laryngeal Cancer diagnosis is still debated.

**Table I**: Imaging modalities commonly used to investigate the spread of laryngeal cancer

| Imaging Modality | Advantages | Drawbacks |
| --- | --- | --- |
| CT | -Accurate assessment of extent of cartilage invasion and submucosal disease.  -5 minutes to capture  -Tolerant to slight movement during scan | -Does not capture vocal cord movement.  -Iodine-enhanced tumours and non-ossified cartilage are challenging to distinguish. |
| MRI | -Useful to determine  pre-epiglottic or paraglottic space invasion. | -Movement of patient during scan can produce blurry images. -Cancer tissue and excessive fluids are challenging to distinguish.  -Can take upto an hour or longer to capture |
| PET CT | -Detects subtle metabolically active lesions | -High chance of false-positives interpretation |
| Ultra-sono-graphy | -Accurate evaluation of paraglottic space involvement  -Non-invasive and non-irradiating | -Not as sensitive as CT or MRI. |

Presentation of a larynx tumor on the same patient on different imaging modalities are very dissimilar as illustrated in Figure 2. Segmentation techniques and their parameters change widely depending on the inputs provided. Conceivably, a segmentation algorithm that works accurately on a given image modality is likely to perform poorly on another. Therefore, it is important to know the features of a particular modality for a given organ and how a tumor presents itself while designing a segmentation technique.


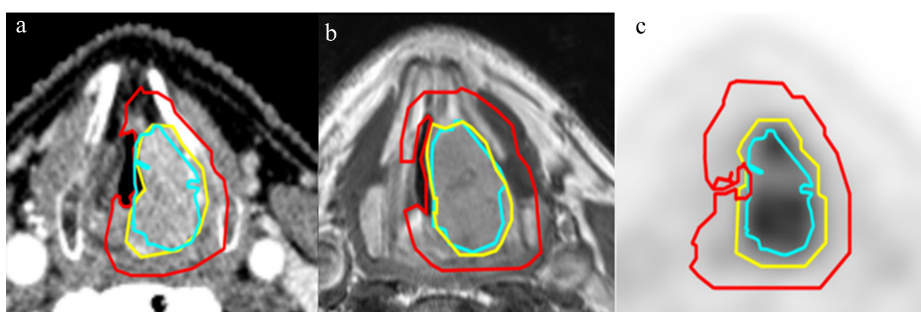


**Figure 2**: Laryngeal tumor delineated on (a) contrast-enhanced CT, (b) MRI and (c) PET CT [8]

This paper focuses on the work that has been done in automating the detection and segmentation of the larynx and tumours in the larynx on CT images. The reason for choosing this modality for this review is that CT remains the more widely used imaging modality. Currently, a standard on imaging for laryngeal cancer imaging does not exist, and the individual cases determine the preference of imaging modality selection [9]. Contrast-enhanced CT are relatively quick to acquire, efficient in cost and computational power required, and tolerant to slight movements during acquisition. CT is often used more widely as it is a readily available and cost efficient option. MR has a greater cost for acquisition in terms of time, computation and resources. Optimization of MR techniques is difficult in this complex part of the body [9]. It is much faster to capture a CT image (60 seconds) while giving comparable results to MRI, which takes longer to capture (30 minutes).; therefore, it requires fewer computing resources compared to an MRI. The short capture time causes clearer images to be obtained and is not affected by patient movements such as coughing, sneezing, breathing etc. [10].

# Importance of Segmentation

### Segmentation is the process of localizing and delineating anatomical structures and tumours in a medical image. Early cancer detection works toward preventing advancement to further stages, thereby increasing chances of a complete recovery. Under-staging a tumour can cause parts of the cancerous tissue to be left behind after treatment. However, if a tumour is over-staged, this could lead to unnecessary loss of healthy tissue, limiting laryngeal function instead of preserving the organ [11]. It is of utmost importance to accurately identify the cancerous tissue and plan the course of treatment action. Segmentation of CT images provides spatial and contextual information that is very valuable as it simplifies analysis and other follow-up tasks such as treatment planning and TNM staging.

### The T stage is dependent on the affected anatomies that may show up only in a single slice, depending on the thickness of the CT image. The size of the tumour and the anatomies affected are crucial pieces of information that determine the stage, which determines the treatment course.

### There are a few challenges in using the CT imaging modality for laryngeal cancer diagnosis. In conventional contrast CT images, the movement of vocal cords is not captured. This is an essential parameter in the T staging of glottic tumours. Mobility of vocal cords has to be observed via clinical reports, or a phonation CT image has to be captured. Iodine-enhanced tumours and non-ossified cartilage are challenging to distinguish. Also, cartilage invasion can cause over-staging as it can appear indistinguishable to the human eye [12].

## Dice Score Coefficient

The Dice Score (DSC) is a widely used metric to evaluate the performance of medical image segmentation methods quantitatively. The segmentation predicted by the image segmentation algorithm (S_Algorithm_) is evaluated against the ground truth segmentation (S_GroundTruth_), as illustrated in Figure 3. High values of DSC are desirable. It addresses class imbalance by penalizing false-positive regions while rewarding accurately segmented regions.


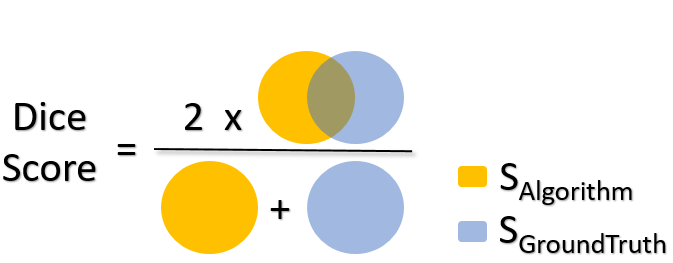


**Figure 3**: Illustration of Dice Coefficient.

# Automated Segmentation Methods

### Automated segmentation of sub-anatomical structures and lesions on the imaging data is useful in saving the detection and diagnostic time of the radiologist and the clinician. Manual outlining of the larynx Region of Interest (ROI) by a radiologist can take up to 2.5 hours for a single CT image series [13].

### There are various approaches to automate the segmentation process.

## Atlas Based Segmentation

Atlas-based methods are widely used in medical image processing for segmentation of anatomy and region of interest [14]. An atlas is a medical image which is pre-labelled with the segmentation and serves as ground truth for the algorithm. When a novel image is to be segmented, it is aligned to the atlas. Alignment is done by identifying vital anatomical structures in the atlas and overlapping the same structures in the novel image, or intensity of pixels, or a variety of other methods with the help of image transformations. After the alignment, a segmentation for the novel image is generated by the segmentation algorithm.

A database of multiple atlases is used instead of a single representative image, to increase the accuracy of segmentation which is known as Multi-Atlas Based Segmentation [15]. The Figure 4 illustrates the segmentation of laryngeal anatomical substructures that was achieved through an atlas based registration technique. Atlas based registration works well for anatomy registration for a given modality due the predictable nature of occurrence unlike presence of malignancies.

*
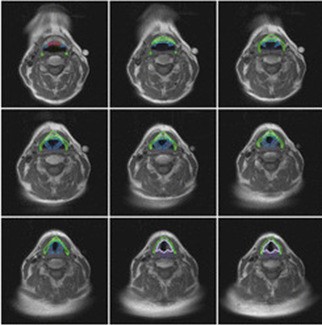
*

**Figure 4**: Atlas-based registration of laryngeal anatomic substructures [16]

## Supervised and Unsupervised Image Segmentation

### Supervised segmentation algorithms use a set of labelled images, which are pre-categorized data, with apriori knowledge and human input for designing the segmentation model. They are quite powerful for automatic segmentation of medical images, albeit not versatile when there is a lot of variance between the train and test sets. They use classification and regression techniques for the segmentation challenge.

### Unsupervised Image segmentation algorithms work with unlabelled data for classification and segmentation. Using the knowledge of the final outcome, they generate a split between non-homogeneous regions into various sub-regions using statistical parameters of the image. Clustering, Association, and Dimensionality reduction are commonly used techniques in this approach. Unsupervised image segmentation needs a larger representative dataset to produce comparable results to supervised methods as they do not have label information. However, the time spent labelling the images is saved as the bulk of the dataset makes up for the lack of detailed input to the model.

## Convolutional Neural Networks

### Convolutional Neural Networks (CNN) are a popular approach that uses Deep Learning for image classification and segmentation. The architecture of a CNN (Figure 5) consists of layers of interconnected nodes with assigned weights that get updated when the model is trained. Pairs of images with the expected segmentations are taken as input to train the model, which learns to segment the ROI by assigning weights to various aspects or objects in the given input images. The final trained model uses these weights to compute the segmentation of the input images. 3D CNNs can capture spatial dependencies on CT images because of the architecture and through the application of relevant filters. Popular CNN architectures include the U-Net, FC-Net and R-CNN which are commonly used for medical image segmentation.


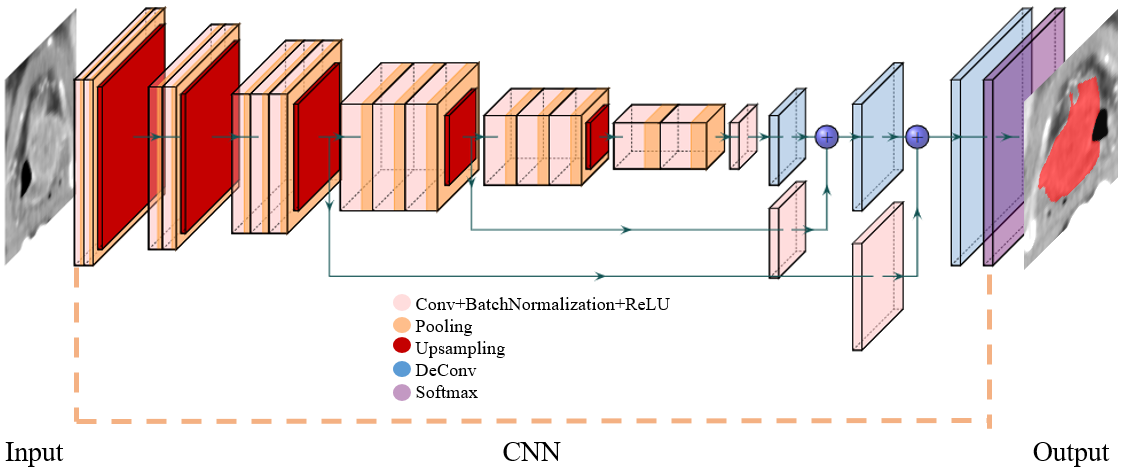


Figure 5: CNN architecture

# Automated Segmentation of the Larynx

The studies reviewed are grouped based on the type of automated segmentation carried out.

## Entire Larynx segmentation

The papers in this subsection worked towards the common goal of segmenting the entire larynx organ at risk from Head and Neck CT images.

Mencarelli et al. [17] designed a hierarchical model to represent substructures with unsupervised learning to detect substructures based on image intensities. The automatic segmentation had an 88% success rate of the time for identifying the ROI of the larynx without manual intervention. They used the Bland Altman method instead of the more straightforward DSC approach for checking the overlap between the expected segmentation and the results obtained. Wu et al. [18] leveraged the relationship hierarchy of detected objects in Head and Neck CT images. They contoured the substructures of Head and Neck CT images with a fuzzy model approach. The DSC varied from 49% to 74%, depending on CT image quality. Tam et al. [31] employed an architecture that exploited shape features and a multiple output support vector model with regression techniques.

Thomson et al. [19] reported results obtained with an atlas-based segmentation approach that was statistically significantly worse than the hierarchical [17] model. Only 8% of the larynx contours could be used as is, without alterations. With the introduction of bias in their algorithm, they could get the DSC significantly higher, to 84%. Another atlas-based approach [20] that used a consensus voting scheme to contour obtained a DSC of 71% for the larynx. Tao et al. [21] used a combination of manual delineation and atlas-based auto segmentation to get a DSC of 73% for the supraglottis and 64% for the glottis segmentation. They aimed to reduce the inter-observer differences during manual contouring resulting in different radiation dosages during treatment. Unbiased contouring could lead to maximum tumour preservation while eliminating tumour tissue.

**Table II**: Summary of Approaches to the Automatic Segmentation of the Larynx using CT images

| Author | Anatomy | DSC | Method | Number of Images |
| --- | --- | --- | --- | --- |
| Tao et al. [21] | Supraglottis | 73% |  | 10 |
| Tao et al. [21] | Glottis | 64% | Atlas- based | 10 |
| Thompson et al. [19] | Larynx | 84% | segmentation | 16 |
| Haq et al. [20] |  | 71% |  | 77 |
| Lei et al. [22] |  | 83% | CNN | 15 |
| Ibragimov et al [23] |  | 85% |  | 45 |
| Willems et al. [24] |  | 39% |  | 90 |
| Liang et al. [25] |  | 87% |  | 185 |
| Zhong et al. [26] |  | 84% |  | 364 |
| Fang et al. [27] |  | 74% |  | 800 |
| Soomro et al [28] |  | 80% |  | 46 |
| van Rooij et al. [29] |  | 78% |  | 136 |
| van Dijk et al. [30] |  | 71% |  | 311 |
| Wu et al [18] |  | 75% |  | 216 |

Ibragimov and Xing [23] were the first to use deep learning methods for the larynx segmentation in Head and Neck CT images. They employed a CNN to identify the consistent intensity patterns and segment the larynx using a 45 image dataset to arrive at an 85.6± 4.2% DSC. Rooij et al. [29] used the 3D U-Net, a deep learning CNN, to contour the larynx ROI. This study used the largest dataset of images we encountered in our review. Their additional initial step of cropping input images around the ROI is not available in the real world scenario. Liang et al. [25] developed a multi-stage CNN network named the ODS-Net. The first detector CNN bounded the larynx with a bounding box. The second CNN used the bounding box to produce a segmentation mask for the larynx. The ODS net is trained on non-contrast CT images and cannot be directly compared to CT with contrast. Zhong et al. [26] used a variation of U-Net for auto contouring the larynx on real-world clinical cases. Though they reached a dice score of 70%, during the oncologists' assessment, the preference was for the manual segmentation compared to the one generated by the CNN. OARNet [28] employs a similar strategy to the ODS Net with multiple CNNs performing detection and segmentation task. With a dense CNN and skip connections, they claimed to increase the accuracy. Lei et al. [22] followed a similar approach with the intent of speed. A Recurrent-CNN quickly detected the larynx with a bounding box while a U-Net segmented it.

## Part Larynx Segmentation

Research work that uses laryngeal CT images for specific sub-anatomy detection and segmentation have been mentioned in this section.

Hewavitharanage et al. [32] developed a supervised support vector machine classifier to segment a single anatomical substructure of the larynx using texture features, as illustrated in Figure 6. The size of the segmented structure is an indicator of vocal cord impairment. With a sample size of 20 subjects, they obtained an 80% DSC score with their ground truth of manual annotations. In another paper by the author [40], an unsupervised image segmentation algorithm was employed to detect the CT slice which contains the vocal fold. They achieved this by localization of the vertebral column and anterior commissure on the CT image. Using a combination of techniques, they achieved an 85% accuracy in estimation in 20 patients. [16]


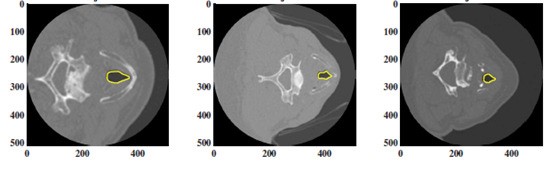


**Figure** **6**: Rimaglottidis segmentation output [32]

## Automated Detection in the Larynx

This section has an overview of detection tasks that were carried out on laryngeal CT images.

Santin et al [34] used deep learning to detect the presence of cartilage abnormalities in CT Scans. With a dataset of 326 images, they obtained an area under the ROC curve of 0.72 in their binary classification problem that used a pre-trained CNN with 83% sensitivity. Lassau et al [35] developed a model that detected tumor invasion in thyroid cartilage. This is a crucial detection task thyroid cartilage invasion worsens the prognosis of laryngeal cancer. They used a dataset of 511 images to train their model, with a result of 70% area under ROC curve. However, the results are not reproducible as details of the model used were not disclosed in the paper. Ayyalu et al. [41] worked on checking the dependence of anatomic similarity for auto-segmentation of Head and Neck CT images. Ten patients captured in different scenarios ranging from poor to perfect used multiple atlases to perform auto contouring. They concluded that auto segmentation of the larynx depended heavily on anatomic similarity compared to the other organs.

# Laryngeal CT Datasets

Data is crucial for designing, testing and validating segmentation algorithms and models. We have curated datasets that contain CT images of the larynx in Table III. The number of images in each dataset, type of segmentation it contains and the availability of the dataset are highlighted.

**Table III** Datasets available

| **Sl. No.** | **No. of Scans** | **Type of CT Scan** | **Segmentation** | **Availability of Dataset** | **Ref.** |
| --- | --- | --- | --- | --- | --- |
| 1 | 45 | Contrast-Enhanced | Larynx | Not Public | [20] |
| 2 | 32 | Contrast-Enhanced |  | Not Public | [20] |
| 3 | 185 | Contrast-Enhanced |  | Not Public | [25] |
| 4 | 1160 | Non-Contrast Enhanced |  | Not Public | [27] |
| 5 | 364 | Contrast-Enhanced |  | On Request | [26] |
| 6 | 265 | Contrast-Enhanced | Tumour | On Request | [33] |
| 7 | 326 | Harmonized slice |  | Not Public | [34] |
| 8 | 606 | Contrast-Enhanced |  | Not Public | [35] |
| 9 | 241 | Contrast-Enhanced |  | Not Public | [36] |
| 10 | 36 | Contrast-Enhanced |  | Public | [37] |
| 11 | 6 | Contrast-Enhanced |  | Public | [38] |
| 12 | 42 | Contrast-Enhanced |  | Public | [39] |

# Commercial contouring software

Commercial contouring software that outlines the larynx ROI for applications such as radiotherapy planning. For the sake of completeness of this review, a thorough comparison has been carried out [13], regarding the efficiency of these algorithms in auto contouring the larynx. Table IV lists the software and their effectiveness. There is scope for the user to manually correct the predictions which enables it to reach a higher efficiency after intervention and validation by human intervention.

**Table IV**: Commercial Auto Segmentation software results on CT images of the Larynx

| **Commercial Software** | **Automatic DSC** | **DSC after manual correction** |
| --- | --- | --- |
| ABAS 2.0 | 86% | 90% |
| MIM 5.1.1 | 87% | 89% |
| Velocity AI 2.6.2 | 82% | 86% |

# Discussion and Conclusion

There have been commendable advances using computer aided techniques for detection, contouring and segmentation on Head and Neck CT images, with promising results. The brain stem, mandible and spinal cord remain the most studied organs at risk. The larynx tends to be side-lined even when the work includes large scale datasets. Recent work with appreciable results that constructed models for automated segmentation of multiple Head and Neck organs at risk [42], [43], [44], [45], [36], [46] did not include the larynx.

There is a lack of a standard for segmentation of the larynx anatomy in CT images, making annotation difficult. Ground truth creation is crucial; maintaining uniformity of annotations across a large dataset is a herculean task. A well-curated representative dataset increases the likelihood that a detection or segmentation model trained on it performs with clinically acceptable sensitivity and specificity. Therefore, it is not easy to develop and validate automated segmentation models.

This review gives an overview on all the computer aided detection and segmentation tasks that have been carried out on laryngeal CT images. A lot of effort has gone towards the segmentation of the larynx as an entire structure, used as region of interest. A few of the studies have focused on segmentation of anatomical substructures and vocal cords.

Our work finds that studies that include laryngeal anatomy for detection or segmentation tasks tend to be limited to a small localized dataset. The availability of segmented laryngeal CT datasets in the public domain is sparse. We hope that our compiled list of available of laryngeal CT images will encourage work in this research gap.

We conclude our paper by highlighting the necessity for research into the laryngeal auto-segmentation, the requirement for the creation of datasets, while highlighting the work done in this field. We argue that there is a great need for more research as standardization and auto segmentation can lead to more effective treatment. If implemented usefully of the larynx, Auto segmentation can be a helpful tool for better, more accessible and quicker diagnosis radiotherapy planning.

# Statements and Declarations

## Funding

This work was supported by Manipal Academy of Higher Education, Dr T.M.A Pai Research Scholarship under Research Registration No. 170100107-2017 and Philips Innovation Campus, Bangalore under Exhibit B-027.

## Ethical Approval

This article does not contain any studies with human participants or animals performed by any of the authors.

## Conflict of Interest

Divya Rao declares that she has no conflict of interest. Dr Prakashini K declares that she has no conflict of interest. Dr Rohit Singh declares that he has no conflict of interest. Vijayananda J declares that he has no conflict of interest.

# References

1. Gupta, B., Johnson, N.W., Kumar, N.: Global epidemiology of Head and Neck cancers: A continuing challenge. Oncology **91**(1), 13–23 (2016). <https://doi.org/10.1159/000446117>
2. Muscat, J.E., Wynder, E.L.: Tobacco, alcohol, asbestos, and occupational risk factors for laryngeal cancer. Cancer **69** (1992). [https://doi.org/10.1002/1097-0142(19920501)69:9h2244:: AID-CNCR2820690906i3.0.CO;2-O](https://doi.org/10.1002/1097-0142(19920501)69:9%3C2244::AID-CNCR2820690906%3E3.0.CO;2-O)
3. Anon: Larynx Gco.iarc.fr (2020). [https://gco.iarc.fr/today/data/ factsheets/cancers/14-Larynx-fact-sheet.pdf](https://gco.iarc.fr/today/data/factsheets/cancers/14-Larynx-fact-sheet.pdf) Accessed 2021-10-08
4. Siegel, R.L., Miller, K.D., Fuchs, H.E., Jemal, A.: Cancer statistics, 2021. CA: A Cancer Journal for Clinicians **71** (2021). [https://doi.org/10.3322/ caac.21654](https://doi.org/10.3322/caac.21654)
5. Anon: Lecturio. 2021. Larynx Concise Medical Knowledge. [https://www. lecturio.com/concepts/larynx/](https://www.lecturio.com/concepts/larynx/)
6. Amin, M., Byrd, D., Edge, S., Greene, F.: AJCC Cancer Staging Manual. Springer, ??? (2016)
7. Issa, M., Samuels, S., Bellile, E., Shalabi, F., Eisbruch, A., Wolf, G.: Tumor volumes and prognosis in laryngeal cancer. Cancers **7** (2015). <https://doi.org/10.3390/cancers7040888>
8. Ligtenberg, H., Jager, E., Caldas-Magalhaes, J., Schakel, T., Pameijer,

F., Kasperts, N., Willems, S., Terhaard, C., Raaijmakers, C., Philippens, M.: Modality-specific target definition for laryngeal and hypopharyngeal cancer on fdg-pet, ct and mri. Radiotherapy and Oncology **123** (2017). <https://doi.org/10.1016/j.radonc.2017.02.005>

1. Junn, J.C., Soderlund, K.A., Glastonbury, C.M.: Imaging of Head and Neck cancer with ct, mri, and us. Seminars in Nuclear Medicine **51** (2021). <https://doi.org/10.1053/j.semnuclmed.2020.07.005>
2. Adolphs, A.P.J., Boersma, N.A., Diemel, B.D.M., Eding, J.E.C., Flokstra,

F.E., Wegner, I., Grolman, W., Braunius, W.: A systematic review of computed tomography detection of cartilage invasion in laryngeal carcinoma. The Laryngoscope **125** (2015). <https://doi.org/10.1002/lary.25145>

1. Li, B., Bobinski, M., Gandour-Edwards, R., Farwell, D.G., Chen, A.M.: Overstaging of cartilage invasion by multidetector ct scan for laryngeal cancer and its potential effect on the use of organ preservation with chemoradiation. The British Journal of Radiology **84** (2011). [https: //doi.org/10.1259/bjr/66700901](https://doi.org/10.1259/bjr/66700901)
2. Kuno, H., Sakamaki, K., Fujii, S., Sekiya, K., Otani, K., Hayashi, R., Yamanaka, T., Sakai, O., Kusumoto, M.: Comparison of mr imaging and dual-energy ct for the evaluation of cartilage invasion by laryngeal and hypopharyngeal squamous cell carcinoma. American Journal of Neuroradiology **39** (2018). <https://doi.org/10.3174/ajnr.A5530>
3. Macchia, M.L., Fellin, F., Amichetti, M., Cianchetti, M., Gianolini, S., Paola, V., Lomax, A.J., Widesott, L.: Systematic evaluation of three different commercial software solutions for automatic segmentation for adaptive therapy in head-and-neck, prostate and pleural cancer. Radiation Oncology **7** (2012). <https://doi.org/10.1186/1748-717X-7-160>
4. Cuadra, M.B., Pollo, C., Bardera, A., Cuisenaire, O., Villemure, J.-G., Thiran, J.-P.: Atlas-based segmentation of pathological mr brain images using a model of lesion growth. IEEE Transactions on Medical Imaging **23**(10), 1301–1314 (2004). <https://doi.org/10.1109/TMI.2004.834618>
5. Iglesias, J.E., Sabuncu, M.R.: Multi-atlas segmentation of biomedical images: a survey. Medical image analysis **24**(1), 205–219 (2015)
6. Choi, M., Refaat, T., Lester, M.S., Bacchus, I., Rademaker, A.W., Mittal, B.B.: Development of a standardized method for contouring the larynx and its substructures. Radiation Oncology **9** (2014). [https://doi.org/10. 1186/s13014-014-0285-4](https://doi.org/10.1186/s13014-014-0285-4)
7. Mencarelli, A., van Kranen, S.R., Hamming-Vrieze, O., van Beek, S., Nico Rasch, C.R., van Herk, M., Sonke, J.-J.: Deformable image registration for adaptive radiation therapy of Head and Neck cancer: Accuracy and precision in the presence of tumor changes. International Journal of Radiation Oncology*Biology*Physics **90**(3), 680–687 (2014). [https: //doi.org/10.1016/j.ijrobp.2014.06.045](https://doi.org/10.1016/j.ijrobp.2014.06.045)
8. Wu, X., Udupa, J.K., Tong, Y., Odhner, D., Pednekar, G.V., Simone, C.B., McLaughlin, D., Apinorasethkul, C., Lukens, J., Mihailidis, D., Shammo, G., James, P., Camaratta, J., Torigian, D.A.: Auto-contouring via automatic anatomy recognition of organs at risk in Head and Neck cancer on CT images. In: Fei, B., III, R.J.W. (eds.) Medical *Article Title*

Imaging 2018: Image-Guided Procedures, Robotic Interventions, and Modeling, vol. 10576, pp. 288–294. SPIE, ??? (2018). [https://doi.org/ 10.1117/12.2293946.](https://doi.org/10.1117/12.2293946) International Society for Optics and Photonics. https://doi.org/10.1117/12.2293946

1. Thomson, D., Boylan, C., Liptrot, T., Aitkenhead, A., Lee, L., Yap, B., Sykes, A., Rowbottom, C., Slevin, N.: Evaluation of an automatic segmentation algorithm for definition of Head and Neck organs at risk. Radiation Oncology **9** (2014). <https://doi.org/10.1186/1748-717X-9-173>
2. Haq, R., Berry, S.L., Deasy, J.O., Hunt, M., Veeraraghavan, H.: Dynamic multiatlas selection-based consensus segmentation of Head and Neck structures from ct images. Medical Physics **46** (2019). [https://doi.org/10.1002/ mp.13854](https://doi.org/10.1002/mp.13854)
3. Tao, C.-J., Yi, J.-L., Chen, N.-Y., Ren, W., Cheng, J., Tung, S., Kong, L., Lin, S.-J., Pan, J.-J., Zhang, G.-S., Hu, J., Qi, Z.-Y., Ma, J., Lu, J.-D., Yan, D., Sun, Y.: Multi-subject atlas-based auto-segmentation reduces interobserver variation and improves dosimetric parameter consistency for organs at risk in nasopharyngeal carcinoma: A multi-institution clinical study. Radiotherapy and Oncology **115** (2015). [https://doi.org/10.1016/ j.radonc.2015.05.012](https://doi.org/10.1016/j.radonc.2015.05.012)
4. Lei, Y., Harms, J., Dong, X., Wang, T., Tang, X., Yu, D.S., Beitler, J.J., Curran, W.J., Liu, T., Yang, X.: Organ-at-Risk (OAR) segmentation in Head and Neck CT using U-RCNN. In: Hahn, H.K., Mazurowski, M.A. (eds.) Medical Imaging 2020: Computer-Aided Diagnosis, vol. 11314, pp. 978–984. SPIE, ??? (2020). [https://doi.org/ 10.1117/12.2549782.](https://doi.org/10.1117/12.2549782) International Society for Optics and Photonics. https://doi.org/10.1117/12.2549782
5. Ibragimov, B., Xing, L.: Segmentation of organs-at-risks in Head and Neck ct images using convolutional neural networks. Medical Physics **44** (2017). <https://doi.org/10.1002/mp.12045>
6. Willems, S., Crijns, W., La Greca Saint-Esteven, A., Van Der Veen, J., Robben, D., Depuydt, T., Nuyts, S., Haustermans, K., Maes, F.: Clinical implementation of deepvoxnet for auto-delineation of organs at risk in Head and Neck cancer patients in radiotherapy, vol. 11041 LNCS, pp. 223–232. Springer, ??? (2018). Stoyanov, D.

*Uhttps* : *//doi.org/*10*.*1007*/*978 − 3 − 030 − 01201 − 4

1. Liang, S., Tang, F., Huang, X., Yang, K., Zhong, T., Hu, R., Liu, S., Yuan, X., Zhang, Y.: Deep-learning-based detection and segmentation

of organs at risk in nasopharyngeal carcinoma computed tomographic images for radiotherapy planning. European Radiology **29** (2019). [https: //doi.org/10.1007/s00330-018-5748-9](https://doi.org/10.1007/s00330-018-5748-9)

1. Zhong, Y., Yang, Y., Fang, Y., Wang, J., Hu, W.: A preliminary experience of implementing deep-learning based auto-segmentation in Head and Neck cancer: A study on real-world clinical cases. Frontiers in Oncology **11** (2021). <https://doi.org/10.3389/fonc.2021.638197>
2. Fang, Y., Wang, J., Ou, X., Ying, H., Hu, C., Zhang, Z., Hu, W.: The impact of training sample size on deep learning-based organ autosegmentation for head-and-neck patients. Physics in Medicine Biology **66** (2021). <https://doi.org/10.1088/1361-6560/ac2206>
3. Soomro, M.H., Nourzadeh, H., Alves, V.G.L., Choi, W., Siebers, J.V.: OARnet: Automated organs-at-risk delineation in Head and Neck CT images (2021)
4. van Rooij, W., Dahele, M., Brandao, H.R., Delaney, A.R., Slotman, B.J., Verbakel, W.F.: Deep learning-based delineation of Head and Neck organs at risk: Geometric and dosimetric evaluation. International Journal of Radiation Oncology*Biology*Physics **104** (2019). [https://doi.org/ 10.1016/j.ijrobp.2019.02.040](https://doi.org/10.1016/j.ijrobp.2019.02.040)
5. van Dijk, L.V., den Bosch, L.V., Aljabar, P., Peressutti, D., Both, S., Steenbakkers, R.J.H.M., Langendijk, J.A., Gooding, M.J., Brouwer, C.L.: Improving automatic delineation for Head and Neck organs at risk by deep learning contouring. Radiotherapy and Oncology **142** (2020). [https: //doi.org/10.1016/j.radonc.2019.09.022](https://doi.org/10.1016/j.radonc.2019.09.022)
6. Tam, C.M., Yang, X., Tian, S., Jiang, X., Beitler, J.J., Li, S.: Automated delineation of organs-at-risk in Head and Neck CT images using multi-output support vector regression. In: Gimi, B., Krol, A. (eds.) Medical Imaging 2018: Biomedical Applications in Molecular, Structural, and Functional Imaging, vol. 10578, pp. 502–511. SPIE, ??? (2018). [https://doi.org/10.1117/12.2292556.](https://doi.org/10.1117/12.2292556) International Society for Optics and Photonics. https://doi.org/10.1117/12.2292556
7. Hewavitharanage, S., Gubbi, J., Thyagarajan, D., Lau, K., Palaniswami, M.: Automatic segmentation of the rima glottidis in 4d laryngeal ct scans in parkinson’s disease. IEEE, ??? (2015). [https://doi.org/10.1109/EMBC. 2015.7318468](https://doi.org/10.1109/EMBC.2015.7318468)
8. Guo, R., Guo, J., Zhang, L., Qu, X., Dai, S., Peng, R., Chong, V.F.H., Xian, J.: Ct-based radiomics features in the prediction of thyroid cartilage invasion from laryngeal and hypopharyngeal squamous cell carcinoma. Cancer Imaging **20** (2020). <https://doi.org/10.1186/s40644-020-00359-2>*Article Title*
9. Santin, M., Brama, C., Th´ero, H., Ketheeswaran, E., El-Karoui, I., Bidault, F., Gillet, R., Teixeira, P.G., Blum, A.: Detecting abnormal thyroid cartilages on ct using deep learning. Diagnostic and Interventional Imaging **100** (2019). <https://doi.org/10.1016/j.diii.2019.01.008>
10. Lassau, N., Estienne, T., de Vomecourt, P., Azoulay, M., Cagnol, J., Garcia, G., Majer, M., Jehanno, E., Renard-Penna, R., Balleyguier, C., Bidault, F., Caramella, C., Jacques, T., Dubrulle, F., Behr, J., Poussange, N., Bocquet, J., Montagne, S., Cornelis, F., Faruch, M., Bresson, B., Brunelle, S., Jalaguier-Coudray, A., Amoretti, N., Blum, A., Paisant, A., Herreros, V., Rouviere, O., Si-Mohamed, S., Marco, L.D., Hauger, O., Garetier, M., Pigneur, F., Berg`ere, A., Cyteval, C., Fournier, L., Malhaire, C., Drape, J.-L., Poncelet, E., Bordonne, C., Cauliez, H., Budzik, J.-F., Boisserie, M., Willaume, T., Moli`ere, S., Faure, N.P., Giurca, S.C., Juhan, V., Caramella, T., Perrey, A., Desmots, F., Faivre-Pierre, M., Abitbol, M., Lotte, R., Istrati, D., Guenoun, D., Luciani, A., Zins, M., Meder, J.-F., Cotten, A.: Five simultaneous artificial intelligence data challenges on ultrasound, ct, and mri. Diagnostic and Interventional Imaging **100** (2019). <https://doi.org/10.1016/j.diii.2019.02.001>
11. Liu, X., Maleki, F., Muthukrishnan, N., Ovens, K., Huang, S.H., P´erezLara, A., Romero-Sanchez, G., Bhatnagar, S.R., Chatterjee, A., Pusztaszeri, M.P., Spatz, A., Batist, G., Payabvash, S., Haider, S.P., Mahajan, A., Reinhold, C., Forghani, B., O’Sullivan, B., Yu, E., Forghani, R.: SiteSpecific Variation in Radiomic Features of Head and Neck Squamous Cell Carcinoma and Its Impact on Machine Learning Models (2021). <https://doi.org/10.3390/cancers13153723>
12. Bosch, W.R., Straube, W.L., Matthews, J.W., Purdy, J.A.: Head-neck cetuximab - the cancer imaging archive (2015). [https://doi.org/10.7937/ K9/TCIA.2015.7AKGJUPZ](https://doi.org/10.7937/K9/TCIA.2015.7AKGJUPZ)
13. Zuley, M.L., Jarosz, R., Kirk, S., Y., L., Colen, R., Garcia, K., Aredes, N.D.: Radiology data from the cancer genome atlas head-neck squamous cell carcinoma [TCGA-HNSC] collection (2016). [https://doi.org/10.7937/ K9/TCIA.2016.LXKQ47MS](https://doi.org/10.7937/K9/TCIA.2016.LXKQ47MS)
14. Brouwer, C.L., Steenbakkers, R.J.H.M., Bourhis, J., Budach, W., Grau, C., Gr´egoire, V., van Herk, M., Lee, A., Maingon, P., Nutting, C., O’Sullivan, B., Porceddu, S.V., Rosenthal, D.I., Sijtsema, N.M., Langendijk, J.A.: Ct-based delineation of organs at risk in the Head and Neck region: Dahanca, eortc, gortec, hknpcsg, ncic ctg, ncri, nrg oncology and trog consensus guidelines. Radiotherapy and Oncology **117** (2015). <https://doi.org/10.1016/j.radonc.2015.07.041>
15. Hewavitharanage, S., Gubbi, J., Thyagarajan, D., Lau, K., Palaniswami,

M.: Estimation of vocal fold plane in 3d ct images for diagnosis of vocal fold abnormalities. IEEE, ??? (2015). [https://doi.org/10.1109/EMBC. 2015.7319049](https://doi.org/10.1109/EMBC.2015.7319049)

1. Ayyalusamy, A., Vellaiyan, S., Subramanian, S., Ilamurugu, A., Satpathy, S., Nauman, M., Katta, G., Madineni, A.: Auto-segmentation of Head and Neck organs at risk in radiotherapy and its dependence on anatomic similarity. Radiation Oncology Journal **37** (2019). [https://doi.org/10.3857/ roj.2019.00038](https://doi.org/10.3857/roj.2019.00038)
2. Zhu, W., Huang, Y., Zeng, L., Chen, X., Liu, Y., Qian, Z., Du, N., Fan, W., Xie, X.: Anatomynet: Deep learning for fast and fully automated whole-volume segmentation of Head and Neck anatomy. Medical Physics **46** (2019). <https://doi.org/10.1002/mp.13300>
3. Vrtovec, T., Moˇcnik, D., Strojan, P., Pernuˇs, F., Ibragimov, B.: Autosegmentation of organs at risk for Head and Neck radiotherapy planning: From atlas-based to deep learning methods. Medical Physics **47** (2020). <https://doi.org/10.1002/mp.14320>
4. Fritscher, K.D., Peroni, M., Zaffino, P., Spadea, M.F., Schubert, R., Sharp, G.: Automatic segmentation of Head and Neck ct images for radiotherapy treatment planning using multiple atlases, statistical appearance models, and geodesic active contours. Medical Physics **41** (2014). [https://doi.org/ 10.1118/1.4871623](https://doi.org/10.1118/1.4871623)
5. Tong, N., Gou, S., Yang, S., Ruan, D., Sheng, K.: Fully automatic multiorgan segmentation for Head and Neck cancer radiotherapy using shape representation model constrained fully convolutional neural networks. Medical Physics **45** (2018). <https://doi.org/10.1002/mp.13147>
6. Nikolov, S., Blackwell, S., Zverovitch, A., Mendes, R., Livne, M., Fauw, J.D., Patel, Y., Meyer, C., Askham, H., Romera-Paredes, B., Kelly, C., Karthikesalingam, A., Chu, C., Carnell, D., Boon, C., D’Souza, D., Moinuddin, S.A., Garie, B., McQuinlan, Y., Ireland, S., Hampton, K., Fuller, K., Montgomery, H., Rees, G., Suleyman, M., Back, T., Hughes, C., Ledsam, J.R., Ronneberger, O.: Deep learning to achieve clinically applicable segmentation of Head and Neck anatomy for radiotherapy (2021)
